# Vitamin D supplementation after the second year of life: joint position of the Committee on Nutrition, German Society for Pediatric and Adolescent Medicine (DGKJ e.V.), and the German Society for Pediatric Endocrinology and Diabetology (DGKED e.V.)

**DOI:** 10.1186/s40348-019-0090-0

**Published:** 2019-05-06

**Authors:** Thomas Reinehr, Dirk Schnabel, Martin Wabitsch, Susanne Bechtold-Dalla Pozza, Christoph Bührer, Bettina Heidtmann, Frank Jochum, Thomas Kauth, Antje Körner, Walter Mihatsch, Christine Prell, Silvia Rudloff, Bettina Tittel, Joachim Woelfle, Klaus-Peter Zimmer, Berthold Koletzko

**Affiliations:** 1 Vestische Kinder- und Jugendklinik Datteln, University Witten/Herdecke, Datteln, Germany; 2 Sozialpädiatrisches Zentrum für chronisch kranke Kinder, Charité Universitätsmedizin Berlin, Berlin, Germany; 3 Children’s Hospital, University of Ulm, Ulm, Germany; 4 LMU, Ludwig-Maximilians-Universität München, Dr. von Hauner Children’s Hospital, Munich, Germany; 5 Neonatology, Charité Universitätsmedizin Berlin, Berlin, Germany; 6 Catholic Children Hospital Wilhelmstift, Hamburg, Pediatric Endocrinology, Clinic Itzehoe, Hamburg, Germany; 7 Evangelisches Waldkrankenhaus Berlin Spandau, Berlin, Germany; 8 Ludwigsburg, Germany; 9 Department of Women’s and Children’s Medicine, Paediatric Research Center, University of Leipzig, Leipzig, Germany; 10 Children’s Hospital, Heliosklinikum Pforzheim, Pforzheim, Germany; 11 Institute for Nutritional Sciences, University of Giessen, Giessen, Germany; 12 Children’s Hospital Dresden-Friedrichstadt, Dresden, Germany; 13 Children’s Hospital, University of Bonn, Bonn, Germany; 14 Children’s Hospital, University of Giessen, Giessen, Germany

**Keywords:** Vitamin D supplementation, Infection, Asthma bronchiale, Hypertension, Obesity, Diabetes mellitus, Attention-deficit/hyperactivity disorder

## Abstract

**Background:**

Low vitamin D serum concentrations have been associated with rickets and other disorders in observational studies. Since vitamin D serum concentrations in children and adolescents are frequently below reference values, it is debated whether vitamin D should be supplemented after infancy.

**Methods:**

The effects of vitamin D supplementation in children > 2 years of age are analyzed based on a literature review of randomized controlled trials (RCTs).

**Results:**

Vitamin D supplementation can potentially reduce the risk for influenza infections and improve asthma bronchiale exacerbation; however, it has no impact on asthma bronchiale severity. Vitamin D supplementation has no relevant effect on attention-deficit/hyperactivity disorders, cardiac failure, hypertension, or incidence of type II diabetes mellitus. Vitamin D supplementation has no effect on the rate of multiple sclerosis relapses, but on the number of new lesions detected by MRI. For other endpoints, RCTs are lacking.

**Conclusion:**

Based on currently available studies, routine vitamin D supplementation is not be recommended for children aged > 2 years, even when they have serum concentrations below reference values. Routine vitamin D supplementation is not recommended in children who do not have risk factors and chronic diseases which are associated with calcium or vitamin D resorption disorders.

## Introduction

The Committee on Nutrition, German Society for Pediatric and Adolescent Medicine, in collaboration with the Pediatric Endocrinology Working Group, wrote a position paper on vitamin D intakes in childhood in 2011 [1, 2]. Metabolism, effects, and intake recommendations for vitamin D were discussed, including the proposition that vitamin D provision promotes age-appropriate mineralization of the skeletal system and prevents the onset of rickets for the first 12–18 months (up until a child’s second early summer) [3, 4]. It has also been shown [2] that subnormal vitamin D serum concentrations (see Table 1 for current definitions) are often measured in children over the age of two [5–8]. In this position statement, a total vitamin D intake of 600 IU/day (from sunlight-dependent self-synthesis and enteral intakes) is considered desirable [2]. The German Nutrition Society recommends a total vitamin D intake of 800 IU/day starting from the age of two [9].

**Table 1:** Recommended limits for vitamin D serum concentrations (25-OH vitamin D) [3]

• > 100 ng/mL (> 250 nmol/L): intoxication	
• 20–100 ng/mL (50–250 nmol/L): target area	
• 12–20 ng/mL (30–50 nmol/L): subnormal	
• < 12 ng/mL (< 30 nmol/L): deficiency	

Since many children in Germany do not achieve desired vitamin D intake levels, it is debated whether and when vitamin D supplementation should take place beyond the first 12 to 18 months of life. Vitamin D not only positively affects the skeletal system but is also postulated to be preventative of inflammatory diseases, autoimmune diseases such as type I diabetes, attention-deficit/hyperactivity disorder, asthma bronchiale, multiple sclerosis, cancers, and lifestyle-related diseases such as obesity, type II diabetes, hypertension, and heart failure [3, 10–14]. However, vitamin D supplementation in children over the age of 2 years of age, even at low vitamin D serum levels, is becoming increasingly controversial [5, 10, 12–14]. Adverse reactions from adequately dosed vitamin D supplements (e.g., 600–800 IU/day) are unlikely, as vitamin D has a broad therapeutic index [2, 3]. However, to make a population-wide recommendation for vitamin D supplementation in childhood and adolescence, a proven benefit should preferably be demonstrated in randomized controlled intervention trials. In addition, existing studies on the relationship between vitamin D and health outcomes must be interpreted together with knowledge of the physiology of vitamin D metabolism, the vitamin D supply in Germany, the possibility of errors in the measurement of vitamin D, and the background of the existing vitamin D target values.

Given the ongoing discussion on when and which children and adolescents should receive vitamin D supplementation after the second year of life, recommendations for vitamin D supplementation for this age range have been updated based on a literature review of randomized controlled trials. 

### Physiology of vitamin D metabolism

Up to 90% of the body’s daily vitamin D requirement is covered by solar radiation (UV-B radiation). The extent of vitamin D synthesis depends on the time of day and the season of the year. Due to Germany’s geographic location (between 48 and 54° northern latitude), UV-B radiation between 10:00 am and 3:00 pm between the months of April and September is sufficient for vitamin D synthesis. However, this is true only if the head and forearms are exposed to the sun without sunscreen for a daily average of about 10–15 min, which is dependent on skin type (skin pigmentation) [2].

About 10% of required vitamin D can be ingested through food. In particular, sea fish such as eel, herring, mackerel, and salmon show relatively high vitamin D contents. From vitamin, 25-OH vitamin D and subsequent calcitriol (1,25 (OH)_2_ vitamin D) are formed via hydroxylation steps in the liver and then in the kidney. Calcitriol regulates calcium and phosphate metabolism and increases the mineralization of the bone.

Vitamin D deficiency leads to calcium deficiency, which results from reduced calcium absorption in the intestine. Low calcium levels in turn lead to an increased parathyroid hormone secretory response. Parathyroid hormone, on the other hand, increases serum calcium levels by increasing the release of calcium from bone and by decreasing calcium excretion into the urine. Rickets manifests itself especially when the daily calcium intake is also low [3].

However, vitamin D not only regulates calcium and phosphate metabolism and thus skeletal mineralization, but also shows multiple modulatory properties in animal experiments, especially on the immune system [15]. Vitamin D regulates more than 300 genes in the mouse model, and the expression of the vitamin D receptor has been detected in about 40 tissues so far [16]. In animal models, after the binding of an infectious agent to the toll-like macrophage receptor, the increased CYP27B1 enzyme stimulation leads to an increased formation of calcitriol. Upon binding to the vitamin D receptor, the formation of cathelicidin and β-defensin 2 is stimulated. Through antiviral, respectively, antibacterial activity, the agent is destroyed [17, 18]. Therefore, it is postulated that vitamin D deficiency could promote the occurrence of autoimmune diseases as well as an increased susceptibility to infections in humans [15].

### Laboratory assessment of vitamin D

Biochemically, vitamin D is a steroid hormone and is as difficult and expensive to determine as testosterone and estradiol. Assessment of vitamin D status is usually conducted by measuring the serum 25-OH vitamin D concentration. The gold standard is HPLC or LC/MS methods and not ELISA, which is the most commonly used method in large laboratories [19]. The advantage of chromatographic or spectrometric methods lies in the high specificity of the detection of vitamin D, whereas in immunoassays, it is very dependent on the antibodies and standards used. To date, there is no internationally recognized standard for vitamin D assessment. Vitamin D is labile to light, so serum tubes must be stored and shipped away from light to avoid false low readings.

### Vitamin D supply in Germany

In the DONALD cohort study, 80% of children aged 1 to 12 years had dietary vitamin D intakes lower than recommended by the German Nutrition Society, based on 3-day dietary protocols [9]. The KIGGS study, which is representative of German children, showed that only 36.1% of children had a vitamin D serum concentration within the target range (see Table 1). Deficiencies were particularly common in winter and spring, in obese children, in children with low physical activity, in children from socially disadvantaged backgrounds, and in children with a migrant background [6, 7]. The high prevalence of vitamin D serum levels below the target range was also confirmed in measurements using the more accurate HPLC method [20].

Low vitamin D serum levels are not only found in Germany but also in other European countries. In a study of 131 healthy adolescents aged 12–15 years in Manchester, more than two thirds of children had vitamin D serum concentrations below the target range in winter and spring and more than one third in summer and autumn [8].

### Interpretation of serum vitamin D levels

Despite the high prevalence of vitamin D serum levels below the target range, overt rickets in infants and adolescents beyond infancy and in the absence of other risk factors (see Table 2) is rare. The recommended target level for vitamin D in children (Table 1) is not adapted for age, gender, or season. If target values were calculated solely based on the statistical distribution of published vitamin D serum concentrations in healthy children and adolescents determined by HPLC, the third percentile would be 8 ng/mL in winter and spring and 14 ng/mL in summer and autumn [8]. However, one cannot conclude that vitamin D concentrations above the third percentile of the distribution achieve adequate health effects.

**Table 2:** Pediatric populations at increased risk for vitamin D deficiency

• Exclusively breastfed infants without vitamin D prophylaxis	
• Infants, children, and adolescents with:	
• Malabsorption or maldigestion disorders (e.g., celiac disease, Crohn’s disease, cystic fibrosis)	
• Chronic inflammatory diseases (e.g., inflammatory bowel disease)	
• Chronic kidney disease	
• Chronic liver disease	
• On permanent medication with substances that affect calcium or vitamin D metabolism (e.g., antiepileptic drugs, antiviral medication, fungicides, or high dose glucocorticoid therapy which inhibits intestinal calcium absorption and stimulates tubular calcium excretion)	
• With very low sun exposure, for example, chronically immobilized children and adolescents	
• With a migrant background (through the influence of pigmentation, nutrition, and sun exposure)	

The recommended vitamin D target values are not based on the normal distribution of apparently healthy populations. Target values are based on considering vitamin D serum levels at which sufficient calcium is absorbed [13, 21, 22]. Considering only enteral vitamin D intakes, healthy people aged 1–70 years of age need a daily intake of 400 IU (estimated average requirement [EAR]), with a mean serum vitamin D level of 16 ng/mL.

**Fig. 1:**
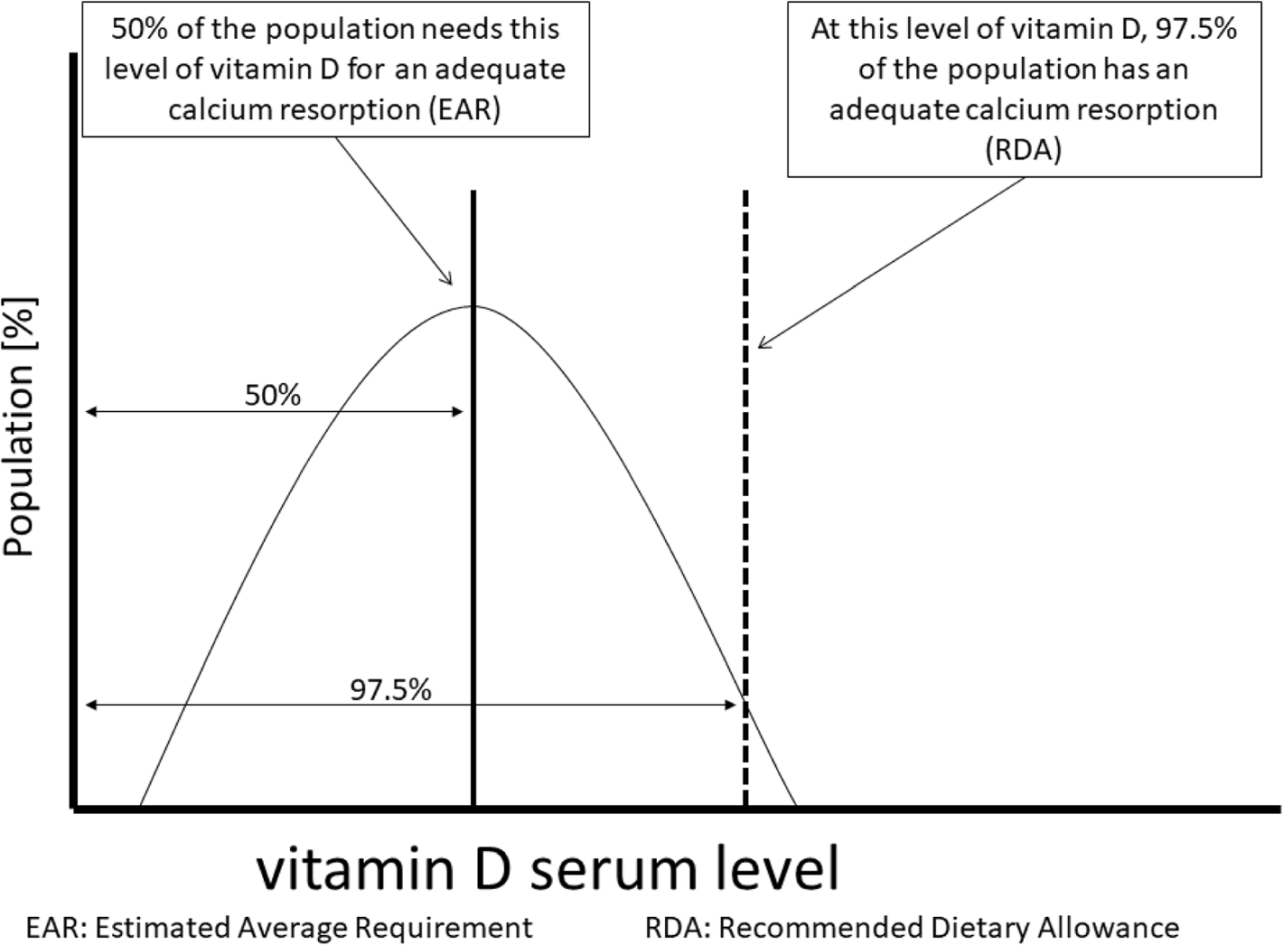
Distribution of vitamin D serum concentrations to ensure adequate calcium absorption in a healthy population and the resulting limits in the absence of sun exposure (figure adapted according to [13])

In order to ensure adequate provision in almost all individuals in a population, a threshold based on the mean plus two standard deviations of the distribution is established, according to the general concepts of nutrient reference levels [23] (see Fig. 1). This results in a targeted vitamin D serum level of 20 ng/mL (50 nmol/L), which corresponds to the Recommended Dietary Allowance (RDA) or the Population Reference Intake (PRI) [24]. However, this limit also implies that many people below this vitamin D level still have adequate calcium absorption [13]. This explains, among other things, why a majority of children with vitamin D serum levels below the target level or even vitamin D deficiency as defined in Table 1 do not develop rickets or apparent alterations in bone mineralization, and only a fraction of these children and adolescents show secondary hyperparathyroidism. In addition, a calcium intake of > 500 mg/day may prevent the manifestation of rickets and subnormal bone mineralization, even at low vitamin D levels [1]. Individual vitamin D therapy is indicated if, in addition to a reduced vitamin D (25-OH vitamin D) concentration, secondary hyperparathyroidism and/or radiologically confirmed rickets are present [3, 5]. A vitamin D serum level below 20 ng/mL does not have any definite pathological significance on its own without the existence of further risk factors [5].

### Vitamin D and skeletal diseases

Rickets, as a vitamin D deficiency disease, as well as its diagnosis and therapy, is discussed in the position statement of DGKJ from 2011 [1, 2] and in international recommendations [3]. In addition to rickets, associations between bone density and vitamin D are also discussed. However, associations alone do not prove causal relationships. For example, the Young Heart 2000 study [25] showed significantly higher forearm bone density at normal vitamin D serum levels in adolescent girls aged 12 to 15 than in girls with vitamin D concentrations below target. In boys, this association was not found. Secondary data analysis revealed that girls’ forearm density was closely associated with cardio-respiratory fitness, which in turn was related to outdoor exercise [26]. Thus, the association between low vitamin D serum levels and forearm bone density could also be indirectly related to outdoor exercise. A low serum level of the “sunshine hormone” vitamin D could be a marker for lack of activity outdoors. This fact also limits the possible deductions from a study describing an association between vitamin D serum concentrations and bone density in adolescent girls [27]. Increased outdoor activity may have positively influenced both vitamin D serum levels and bone mineral density. In fact, the same authors conclude that increased physical activity increases bone density [28]. In a randomized controlled trial, however, a positive effect of vitamin D on the bone density of girls could be demonstrated. This effect was no longer statistically significant in an intention-to-treat analysis including girls who did not take the vitamin D supplement on a regular basis [29]. It was concluded from meta-analyses of placebo-controlled trials that vitamin D supplementation in healthy children does not improve bone density, but may have positive effects in children with vitamin D serum concentrations below target [30, 31].

### Vitamin D and extraskeletal diseases

Vitamin D deficiency is not only associated with skeletal disorders [3], but also with various other diseases [15] (see Table 3). Most of the postulated correlations are based on association studies or epidemiological studies. Controlled randomized intervention studies are often lacking or fail to show any effect of vitamin D on the respective diseases examined in the case of the few available intervention studies (see Table 3).

**Table 3:** Pediatric and adolescent diseases that were postulated to be associated with vitamin D serum concentrations due to their associations in observational studies

	References	Effect of vitamin D supplementation from RCTs
Diseases of the upper airway	[10, 14, 37–40, 51]	+ and −
Asthma bronchiale	[36, 52, 53]	+ and −
Attention-deficit/hyperactivity disorder	[54–56]	−
Type I diabetes mellitus	[10, 14, 32–35]	No RCT performed
Type II diabetes mellitus	[10, 14, 44–50]	+ and −
High blood pressure	[10, 14, 41, 43]	−
Cardiac insufficiency	[10, 14]	−
Obesity	[10, 11, 14, 57–59]	No RCT performed
Multiple sclerosis	[60–63]	+ and −*

*RCT* randomized controlled trials, + RCT showed a positive effect from vitamin D supplementation on the studied parameter, − RCT showed no effect from vitamin D supplementation on the studied parameter

*Vitamin D had no effect on the frequency of multiple sclerosis relapses but a positive effect on the number of sclera on MRI

Another example of the difficulties in interpreting association studies is the postulated association between vitamin D and the prevalence of type I diabetes mellitus (T1DM). In Finland, a dose reduction of vitamin D supplementation from 2000 IU/day in 1965 to 400 IU/day in 1995 was associated with an increased prevalence of T1DM with [32]. No further increase in T1DM incidence occurred since 2006 [33]. Therefore, it was postulated that vitamin D supplementation could reduce the risk of developing T1DM by up to 30% [34]. However, serum vitamin D levels before initiation of seroconversion of islet cell autoantibodies or prior to manifestation of T1DM did not differ from those in children who did not have T1DM and had no islet cell autoantibodies [35]. This suggests that a causal relationship between vitamin D serum concentrations and the risk of developing T1DM is questionable.

A positive effect of vitamin D supplementation on childhood asthma bronchiale was shown in randomized controlled trials. Vitamin D supplementation in children with asthma bronchiale has led to a significant reduction in asthma bronchiale exacerbations and the frequency of inpatient hospital admission in children with mild asthma bronchiale [36]. However, the forced expiratory volume in one second (FEV1) as a measure of disease severity did not improve with vitamin D supplementation in randomized controlled trials [36].

The effect of vitamin D on asthma bronchiale is attributed to a reduction in the frequency of acute upper respiratory infections [36]. In a randomized controlled trial, 169 children aged 6 to 12 years received 1200 IU of vitamin D per day during the winter months, while 137 children of the same age received placebo [37]. As a result, the prevalence of influenza A infection in the intervention group was significantly lower. Another randomized controlled trial showed that in school children between the ages of 8 and 12 years, the number of parent-reported upper respiratory diseases in the intervention group (intake of 300 IU of vitamin D-enriched milk in the winter months) was significantly lower than that in children without vitamin D supplementation [38].

Another randomized study in 354 children aged 1 to 5 years showed no difference between higher-dose vitamin D supplementation (2000 IU/day) and 400 IU/day vitamin D supplementation for viral upper airway infections during winter months [39]. The authors of a recent Cochrane Review based on four randomized controlled trials on nearly 3200 children in Afghanistan, Spain, and the USA concluded that a protective effect from vitamin D supplementation on pneumonia and diarrhea risk in children up to 5 years age has not been established [40].

Randomized controlled trials did not show any significant or clinically relevant effects of vitamin D on other diseases. A meta-analysis of studies on vitamin D supplementation predominantly in adults found no evidence for a preventative effect on cancer or lifestyle-related diseases [10, 14]. Meta-analyses from randomized controlled trials showed that vitamin D supplementation in arterial hypertension resulted in a small reduction in diastolic blood pressure (by 2 mmHg) but not in a reduction in systolic blood pressure [41]. Vitamin D supplements did not result in an improvement in vascular status [42] or cardiovascular performance [43]. While association studies postulate a possible effect of vitamin D supplementation on fasting glucose, HbA1c, and insulin resistance in type II diabetes mellitus [44], randomized controlled trials have shown no influence of vitamin D supplements on the rate of progression from prediabetes to type II diabetes mellitus [45] on the risk of developing type II diabetes mellitus at low vitamin D concentrations [46], glucose-insulin metabolism [47], or insulin resistance in children [48]. According to current meta-analysis of randomized controlled trials, in patients with type II diabetes, vitamin D supplementation positively affected HbA1c levels in patients with low vitamin D concentrations [49] or with high dose vitamin D supplements (4000 IU/day) [50].

### Conclusions and recommendations

- During childhood and adolescence, adequate vitamin D status is desirable to promote enteral calcium absorption and thus bone health.
- In addition to the vitamin D provided in breast milk or infant formula, an oral vitamin D3 supplement (400–500 IU/day) is recommended for all infants in Germany until their second early summer. Depending on the time of their birth, vitamin D supplementation is recommended for a period of one to one and a half years, since during summer, increased ultra violet exposure and vitamin D self-synthesis occurs. A combined supplement comprised of vitamin D and fluoride prophylaxis should be given.
- The total daily intake of vitamin D for premature infants weighing less than 1500 g at birth is 800–1000 IU/day in the first few months of life.
- A desirable total vitamin D intake (from sunlight-dependent, endogenous synthesis and enteral intake) for children older than 1 year, adolescents, and adults is 600–800 IU/day.
- Regular outdoor activities for children not only improve vitamin D synthesis, but also lead to further positive health effects. Exposure to sunlight improves vitamin D status while exercise increases bone mass development. Exposure to sunlight between April and September, at least twice a week between 10:00 am and 3:00 pm for 5 to 30 min is the most effective form of improving vitamin D status. Playing outside with an uncovered head, arms, and legs is adequate for vitamin D production in children and adolescents with skin types II and III. Care should be taken to avoid sunburn.
- Children and adolescents should regularly (once or twice a week) consume fish rich in vitamin D. Fish consumption is desirable for various reasons and contributes to vitamin D intakes.
- There are indications that vitamin D supplementation has possible preventive effects on the risk for infections. However, these effects are not proven beyond doubt.
- Untargeted testing of vitamin D serum concentrations in healthy children without risk factors for vitamin D deficiency is not recommended. Groups at high risk of vitamin D deficiency include children and adolescents with certain chronic diseases and risk factors (Table 2). In these cases, prophylactic vitamin D supplementation (500–1000 IU/day) may be useful, especially in the winter months.
- A 25-hydroxy-vitamin D serum concentration (< 20 ng/ml) does not constitute an indication for vitamin D supplementation unless there are additional risk factors (Table 2).
